# First HIV prevalence estimates of a representative sample of adult sub-Saharan African migrants in a European city. Results of a community-based, cross-sectional study in Antwerp, Belgium

**DOI:** 10.1371/journal.pone.0174677

**Published:** 2017-04-05

**Authors:** Jasna Loos, Christiana Nöstlinger, Bea Vuylsteke, Jessika Deblonde, Morgan Ndungu, Ilse Kint, Lazare Manirankunda, Thijs Reyniers, Dorothy Adobea, Marie Laga, Robert Colebunders

**Affiliations:** 1 HIV and Sexual Health Unit, Department of Public Health, Institute of Tropical Medicine, Antwerp, Belgium; 2 Epidemiology of Infectious Diseases Unit, Department of Public Health and Surveillance, Scientific Institute of Public Health, Brussels, Belgium; 3 Community researcher of the TOGETHER Project, HIV and Sexual Health Unit, Department of Public Health, Institute of Tropical Medicine, Antwerp, Belgium; 4 AIDS Reference Center, Department of Clinical Sciences, Institute of Tropical Medicine, Antwerp, Belgium; 5 Epidemiology for Global Health Institute, Faculty of Medicine & Health Sciences, University of Antwerp, Antwerp, Belgium; Indiana University, UNITED STATES

## Abstract

**Introduction:**

While sub-Saharan African migrants are the second largest group affected by HIV in Europe, sound HIV prevalence estimates based on representative samples of these heterogeneous communities are lacking. Such data are needed to inform prevention and public health policy.

**Methods:**

This community-based, cross-sectional study combined oral fluid HIV testing with an electronic behavioral survey. Adopting a two-stage time location sampling HIV prevalence estimates for a representative sample of adult sub-Saharan African migrants in Antwerp, Belgium were obtained. Sample proportions and estimated adjusted population proportions were calculated for all variables. Univariable and multivariable logistic regression analysis explored factors independently associated with HIV infection.

**Results:**

Between December 2013 and October 2014, 744 sub-Saharan African migrants were included (37% women). A substantial proportion was socially, legally and economically vulnerable: 21% were probably of undocumented status, 63% had financial problems in the last year and 9% lacked stable housing. Sexual networks were mostly African and crossed national borders, i.e. sexual encounters during travels within Europa and Africa. Concurrency is common, 34% of those in a stable relationship had a partner on the side in the last year. HIV prevalence was 5.9%(95%CI:3.4%-10.1%) among women and 4.2% (95%CI:1.6%-10.6%) among men. Although high lifetime HIV testing was reported at community level (73%), 65.2% (CI95%:32.4%-88.0%) of sub-Saharan African migrants were possibly undiagnosed. Being 45 years or older, unprotected sex when travelling within Europe in the last year, high intentions to use condoms, being unaware of their last sexual partners’ HIV status, recent HIV testing and not having encountered partner violence in the last year were independently associated with HIV infection in multivariable logical regression. In univariable analysis, HIV infection was additionally associated to unemployment.

**Conclusions:**

This is the first HIV prevalence study among adult sub-Saharan African migrants resettling in a European city based on a representative sample. HIV prevalence was high and could potentially increase further due to the high number of people with an undiagnosed HIV infection, social vulnerability, high levels of concurrency and mainly African sexual networks. Given this population’s mobility, an aligned European combination prevention approach addressing these determinants is urgently needed.

## Introduction

Migrants from high endemic regions, including those from sub-Saharan Africa are the second largest group affected by HIV/AIDS in the European Union/European Economic Area (EU/EEA) and a priority group for HIV prevention. In 2014, 13.8% of new HIV diagnoses in EU/EEA Member states were among sub-Saharan African migrants [1]. Since 2005, surveillance data show a decreasing trend of HIV diagnoses among this group [1, 2]. It is unclear how to interpret this trend. Potential explanations refer to a truly decreasing incidence, changes in migration flows or lower uptake of HIV testing as a result of toughening immigration laws and restricted access to health care and social rights [2]. Consequently, surveillance data provide little indications for prevention planning. While there is a pressing need for tailored and targeted interventions aiming to prevent new HIV infections since increasing evidence is showing that sub-Saharan African migrants are acquiring HIV in Europe [3].

HIV prevalence estimates could serve as a complementary indicator of the HIV burden, besides the numbers of newly reported HIV diagnoses, and could guide prevention planning. Currently such HIV prevalence estimates are only available for the United Kingdom: mathematical modelling estimated that among 15–44 year old black heterosexual Africans 17.9 per 1,000 men and 43.7 per 1,000 women were living with HIV in 2014 [4]. For other European countries few HIV prevalence estimates are available, but they are not representative. The Mayisha II study, conducted in 2004 in London, Luton and the West-Midlands reported an overall prevalence of 14%, 15% among women and 13.1% among men. This study might have oversampled HIV positive people by using a convenience sample of recruitment sites [5,6]. In other prevalence studies, sub-Saharan African migrants were a sub-group amongst other target groups, such as migrant female sex workers [7], recently arrived migrants [8], attendees of a tropical medicine referral unit [9] or were studied in combination with other ethnic minorities [10].

Several reasons may account for the lack of representative HIV prevalence estimates for sub-Saharan African migrant residing in Europe. First, sub-Saharan African migrant communities are small but heterogeneous in terms of their national, ethnic and cultural backgrounds, migration patterns, residence status, educational and socio-economic backgrounds, and religious beliefs [11]. These different communities all have their particular characteristics and distinct meeting places, which makes it difficult for obtaining a sound representative sample of sub-Saharan African migrants. Second, researchers and funders may have been reluctant to conduct such studies due to concerns of reiterating the persisting HIV related stigma and discrimination among these communities and further contributing to general xenophobia [11,12]. Finally, because the HIV epidemic among migrants in Europe was long considered an imported one, research and prevention efforts were mostly focused on early linkage to care through promotion of HIV testing and counseling [3, 12]. This perception recently changed with increasing evidence of HIV acquisition in Europe [3]. The aMASE study, conducted in nine European countries, showed that 31% of HIV infected Africans acquired HIV while living in European host countries, underlining the renewed need for primary prevention [13]. To inform interventions aiming to prevent new HIV infections among sub-Saharan African migrants in Europe, HIV prevalence studies are needed to understand the magnitude of the epidemic and the risk for onwards transmission.

As for Europe in general, the HIV epidemic among sub-Saharan African migrants in Belgium, the country of our study, has not been well described. People with a sub-Saharan African nationality accounted for 29% of new HIV diagnoses reported in 2014, 30% less than in 2012 [14]. It is unclear whether this reflects a declining epidemic, reduced HIV testing uptake, or changing migration patterns [2]. HIV testing uptake may have been affected by the increased restrictions to access health care for undocumented migrants [15]. However, the number of late HIV diagnoses (at < 350 CD4/mm^3^) among patients with a sub-Saharan African nationality remained stable at around 50% in the last years [14]. The impact of restrictive migration laws on HIV surveillance trends [2], and the number of undiagnosed HIV are difficult to establish, since accurate population size estimates of sub-Saharan African migrants are not available. Officially 175,000 people who are born in a sub-Saharan African country are registered in Belgium. This is an underestimation, as people of undocumented status, sub-Saharan African migrants who obtained Belgian nationality, and second and third generation migrants are not included in this number. Also the increased mobility of migrants since the recent economic crisis is not accounted for in official population numbers [15]. Sub-Saharan African migrants who moved from other European countries to Belgium in search for job opportunities quickly become part of the local sexual networks [16], and this may also influence the Belgian HIV epidemic. Therefore, the present study’s objective was to obtain sound HIV prevalence estimates and to identify sub-groups that should be prioritized for HIV prevention through studying a representative sample of the heterogeneous sub-Saharan African migrant communities present in Antwerp city (i.e. the largest Flemish city

## Methods

We conducted a cross-sectional community-based survey combining biological and behavioral data using a two-stage time location sampling. The study design was informed by three formative sub-studies and adopted a community based participatory research approach [17,18,19].

### Community based participatory research approach

To assure acceptability, a Community Advisory Board was set up and a team of community researchers engaged. This team of nine lay people of sub-Saharan African origin reflected the communities’ diversity in terms of origin, educational background and migration status. In line with GIPA principles [20], sub-Saharan African migrants living with HIV were also included in the team. They were involved in every step of the research process and received training and continuous coaching from the first author throughout the study to ensure data rigor [18].

### Study population and sampling frame

Officially about 17,000 people of sub-Saharan African origin are living in Antwerp city, which, however, excludes people of undocumented status and mobile groups. About 11,000 of them are adults (i.e. 18 years or older) and almost half (47%) originate from three countries: The Democratic Republic of the Congo (18.8%), Ghana (17.5%) and Nigeria (10.7%). In addition, 43 other nationalities are living in the Antwerp city area.

To include a representative sample of the diverse communities of sub-Saharan African migrants, including hard to-reach groups like undocumented migrants and mobile populations, we opted for time location sampling. This method takes into account that some hard-to-reach populations tend to gather and can be reached at certain types of locations [21]. To construct the sampling frame we conducted a formative study. Between June 2012 and June 2013, community settings frequented by sub-Saharan African migrants in Antwerp city were systematically mapped using an adaptation of the PLACE Method Approach [17]. The community researchers interviewed a total of 223 adults knowledgeable about the communities’ social meeting places. After reaching saturation, community researchers visited all identified sites for a verification interview. We assessed the busiest moments, and the number of people usually present during these times per site to allow proportion-to-size sampling. The obtained sampling frame consisted of a list of 169 community settings: bars, churches, events and meetings of African organisations, public places (like squares, parks and street corners), hairdressing salons and shops.

### Sample size and sampling method

The sampling size was calculated to detect an HIV prevalence of 4%, with a precision of 2% and a type I error (alpha) of 0.05. We assumed the study design effect of the cluster sampling to be 2, resulting in a required sample size of 714 sub-Saharan African migrants.

A two-stage time location sampling was used to recruit the required number of study participants. First, 51 clusters or settings, were selected with a probability proportion-to-size from the sampling frame. If a selected setting was unavailable or declined, we documented this and selected the next setting on the list. In a second step we randomly selected 14 participants present at the setting at the time of data collection. If the target of 14 participants could not be reached at the first visit, up to three study visits were made to the same setting until the desired number of participants was achieved.

### Inclusion criteria

All people socializing in a given setting at the time of the study visit were eligible for inclusion if they met the following criteria: (1) self-identified sub-Saharan African migrant, (2) age 18 years or above, (3) accepting to answer the questionnaire, (4) accepting to donate an oral fluid sample, (5) providing written informed consent. Each person could participate only once in the study.

### Data collection procedures

Standard Operating Procedures (SOPs) were developed in collaboration with the community researchers, the community advisory board and the AIDS reference laboratory at the Institute of Tropical Medicine. These SOPs were refined after two pilots. At pre-arranged moments the study team visited the settings and randomly selected 14 persons. The procedures were setting-specific depending on type of venue. For instance, in churches each community researcher was assigned randomly to a row of chairs and they invited everybody sitting in that row to participate. In bars or at meetings, tables were assigned at random. At parties, public events or in public places, randomly assigned walking lines were followed. In settings with few people present, everybody was selected.

The community researchers presented themselves to potential study respondents, introduced the study, its objectives and methods stressing anonymity and voluntary nature of participation. Community researchers explicitly mentioned that everybody was invited to participate, regardless of their HIV status in order to avoid self-exclusion of HIV positive people.

People interested to participate received more detailed information on the procedures and were asked to sign the informed consent form. Upon signing, they self-completed an electronic behavioral questionnaire (surveyTo Go, www.dooblo.net) on a 7-inch tablet. To build confidence and ensure data rigor, a short tutorial on how to handle the tablet was integrated. It gave special attention to anonymizing aspects of the data collection. If needed, community researchers offered discrete assistance to fill in the questionnaire. Next, participants were asked to self-collect an oral fluid sample for HIV testing. The collected samples were put in a dry tube and stored in a closed envelope together with the other data sources. A unique code linked the informed consent, questionnaire, oral fluid sample, and a result recollection letter. Participants could obtain their HIV test result by calling the study nurse and providing their unique study code if they wanted to learn their results.

Finally, community researchers collected attendance data. They asked Participants in a uniform way how often they had visited the study setting in the last month. As a token of appreciation participants received free condoms, an information brochure on HIV testing and € 5,-. Those who refused received the same, except for the financial compensation. Limited data on decliners’ sociodemographic characteristics and reasons of refusal were recorded. Since it was not feasible to ask decliners about their age or country of origin, these data were provided based on community researchers’ assumptions.

### Laboratory methods

The main outcome measure was HIV status. It was established using the oral fluid specimens. Within seven days of collecting the sample, the AIDS reference laboratory performed the analysis according to a validated algorithm [22]. First, samples were tested with a Genscreen HIV ½ v2® (BioRad, Sensitivity = 100, 95% CI: 95.9–100; Specificity = 97.6, 95% CI: 94.5–99.0). If reactive, a second HIV ELISA test, Vironostika HIV Ag/Ab (BioMérieux, Sensitivity = 97.8, 95% CI: 92.3–99.4; Specificity = 100, 95% CI: 98.2–100) was performed. Only participants with two reactive test results were considered to be HIV infected. The quality of the oral fluid samples was measured for all negative samples using an IgG ELISA quantification kit (Human Total IgG ELISA, Immunology Consultants Laboratory, Inc., cat. No: E-80G). Samples with insufficient IgG were considered non-valid and excluded from analysis.

### Statistical analysis

All data were merged, cleaned and analyzed using SPSS Statistics 23 (SPSS Inc., Chicago, US). In a first step we calculated simple sample proportions of relevant variables. Next, we estimated adjusted population proportions [23] using the complex samples module of SPSS. This module allowed us to account for the design specifications of time-location sampling, e.g. unequal probability of selection and clustering (e.g. individuals selected from the same venue tend to behave in a similar way) [24]. To account for the unequal probability of selection, weighting factors were calculated taking into account the probability that a given participant was in the setting at the time of the study (calculated based on the attendance data, see above), the probability of accepting participation taking into account age and setting (obtained from a logistical model with participation as dependent variable and age and setting as independent variables) and the sampling fraction (e.g. number of participants/ total number of sub-Saharan African migrants present in the study setting at the time of the study) [24]. To account for the cluster design characteristics in the point estimates, variances of parameter estimates were computed according to the Taylor linearization method as presented by Binder [25]. The study venue was defined as the unit of clustering. Both weighting factors and clusters were included into the SPSS Complex samples plan file to estimate population proportions and 95% confidence intervals (CI) of socio-demographic characteristic, migration factors, sexual behavior and sexual health. For these variables, a Chi-square was calculated to analyze for differences in gender.

The SPSS Complex samples module and plan file was also used to conduct a univariable and multivariable analysis. HIV status was considered the dependent variable. Independent variables included socio-demographic variables, migration, sexual behavior and sexual health factors. The strength of the association was measured by the Odds ratio. Variables associated in univariable analysis with *p*<0.1 were included in the multivariable logistic regression analysis. A *p*<0.05 in multivariate analysis was considered statistically significant.

### Ethical approval

Ethical approval was obtained of the Institutional Review Board of the Institute of Tropical Medicine and the Ethical committee of the University Hospital Antwerp.

## Results

**Fig 1:**
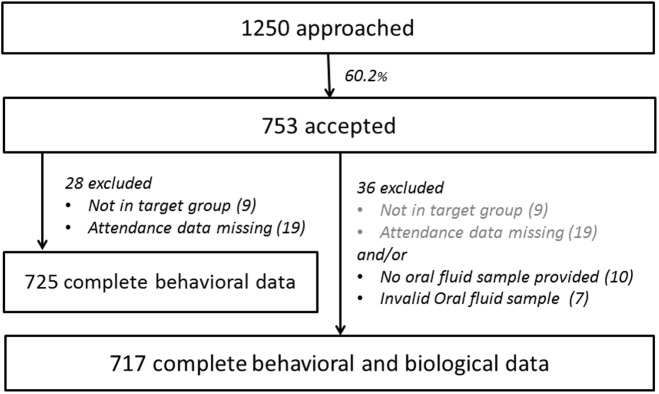
Flowchart of cases included in the unweighted and weighted analysis, generating respectively sample- and population estimates.

Between December 13^th^, 2013 and August 31^st^, 2014 we conducted 77 data collection visits to 51 cluster settings spread over nine districts of Antwerp city. This included fourteen activities of socio-cultural organizations of sub-Saharan African migrants, twelve churches, ten bars, seven public places, two shops, two hairdressing salons, an asylum center and a public library. In total, 1250 potential participants were approached, of whom 753 accepted (see Fig 1). A non-response analysis showed no differences regarding gender between those who participated and those who declined. People of older age were more likely to decline participation than those estimated to be 18–25; and those approached in churches or African organization meetings were more likely to participate when compared with those approached in public spaces. Not having time was the most frequently mentioned reason for non-participation. Below the population estimates of 725 participants are presented.

### Socio-demographic and migration related characteristics

Selected background characteristics are shown in Tables 1 and 2. More men (63.1%) than women participated in the study. Participants were between 18 and 82 years old, with a mean age of 34.5 years. Seventy-nine percent were residents of Antwerp city.

**Table 1:** Socio- demographic characteristics of the sample (unweighted) and population estimates of sub-Saharan African migrants socializing in Antwerp city (weighted), stratified by gender.

	TOTAL		FEMALE		MALE		P-value
	Sample	Population	Sample	Population	Sample	Population	Population
	% (N = 744)	% (CI 95%)	% (N = 314)	% (CI 95%)	% (N = 430)	% (CI 95%)	
**Study setting**							
*Bar/party of African organization*	39.9% (297)	52.4% (33.8%-70.3%)	34.4% (108)	41.6% (25.1%-60.2%)	44.0% (189)	58.7% (38.4%-76.4%)	.008*
*Church*	23.3% (173)	23.5% (11.1%-42.9%)	29.9% (94)	32.4% (16.7%-53.3%)	18.4% (79)	18.3% (7.8%-37.2%)	
*Public place (park*, *street*, *square)*	14.5% (108)	11.7% (5.1%-24.4%)	10.5% (33)	11.3% (4.3%-26.5%)	17.4% (75)	11.9% (5.1%-25.4%)	
*Meeting of African organization*	9.8% (73)	5.0% (1.8%-13.1%)	13.4% (42)	7.5% (2.7%-19.0%)	7.2% (31)	3.6% (1.1%-11.1%)	
*Other (shop*, *hair salon*, *library*, *asylum center)*	12.5% (93)	7.5% (3.0%-17.6%)	11.8% (37)	7.3% (2.9%-17.1%)	13.0% (56)	7.6% (2.7%-19.6%)	
**Age**							.047*
*18–24 years*	19.3% (144)	17.8% (12.2%-25.1%)	23.2% (73)	22.9% (13.6%-35.9%)	16.5% (71)	14.8% (10.2%-20.9%)	
*25–34 years*	37.0% (275)	35.3% (29.6%-41.5%)	41.4%(130)	39,9% (31.7%-48.7%)	33.7% (145)	32.7% (25.8%-40.3%)	
*35–44 years*	27.0% (201)	28.8% (23.8%-34.4%)	23.2% (73)	22,4% (15.5%-31.2%)	29.8% (128)	32.6% (26.5%-39.3%)	
*≥ 45 years*	16.7% (124)	18.1% (14.2%-22.7%)	12.1% (38)	14,8% (9.2%-23.0%)	20.0% (86)	20.0% (15.0%-26.0%)	
**Living in Belgium**	95.6% (711)	94.6% (91.1%-96.8%)	94.3% (296)	93.9% (88.0%-97.0%)	96.5% (415)	95.0% (90.8%-97.3%)	.628
**Educational level**^a^ ^b^							.013*
*Primary school or less*	17.6% (128)	16.0% (11.9%-21.3%)	23.1% (71)	21.2% (13.6%-31.6%)	13.6% (57)	12.9% (9.0%-18.2%)	
*Completed secondary*	47.5% (345)	49.1% (43.3%-54.9%)	48.5% (149)	53.7% (42.0%-65.0%)	46.8% (196)	46.3% (40.6%-52.1%)	
*Continued education*	34.8% (253)	34.9% (29.1%-41.2%)	28.3% (87)	25.0% (17.6%-34.3%)	39.6% (166)	40.8% (34.4%-47.5%)	
**Occupation**^a^							.026*
*(Self-)employed*	45.9% (325)	47.6% (41.7%-53.6%)	40.4% (122)	38.4% (31.0%-46.4%)	50.0% (203)	53.2% (44.7%-61.5%)	
*Unemployed*	43.5% (308)	42.2% (36.6%-48.0%)	45.0% (136)	48.0% (39.7%-56.4%)	42.4% (172)	38.7% (30.8%-47.3%)	
*Full time student*	10.6% (75)	10.2% (7.1%-14.3%)	14.6% (44)	13.6% (8.4%-21.3%)	7.6% (31)	8.1% (5.3%-12.2%)	
**Financial problems in the last year**^a^							.357
*Most of the time*	24.5% (173)	26.6% (21.7%-32.1%)	21.6% (64)	22.0% (15.8%-29.7%)	26.5% (109)	29.2% (22.4%-37.1%)	
*Sometimes*	38.8% (274)	36.9% (31.9%-42.2%)	39.5% (117)	39.5% (32.0%-47.6%)	38.2% (157)	35.3% (28.1%-43.3%)	
*No*	36.8% (260)	36.6% (32.3%-41.1%)	38.9% (115)	38.5% (30.2%-47.5%)	35.3% (145)	35.4% (30.7%-40.4%)	
**Unstable housing***^c^*	10.7% (60)	8.8% (5.7%-13.3%)	12.8% (30)	11.1% (5.9%-20.1%)	9.2% (30)	7.4% (4.2%-12.9%)	.341

^a^ Prefer not to answer was excluded from analysis: educational level (N = 726), occupation (N = 708), financial problems (N = 707).

^b^ Highest level of schooling completed.

^c^ Unstable housing includes those who are homeless or living with friends (N = 559).

* Significant difference between men and women at 5% level.

**Table 2:** Migration related characteristics of the sample (unweighted) and population estimates of sub-Saharan African migrants socializing in Antwerp city (weighted), stratified by gender.

		TOTAL		FEMALE		MALE		P-value
		Sample	Population	Sample	Population	Sample	Population	Population
		% (N = 744)	% (CI 95%)	% (N = 314)	% (CI 95%)	% (N = 430)	% (CI 95%)	
**Region of origin**								.097
*Western Africa*		66.7% (496)	66.3% (52.1%-78.1%)	73.9% (232)	72.0% (58.0%-82.6%)	61.4% (264)	63.0% (47.1%-76.5%)	
*Central Africa*		26.3% (196)	27.8% (17.5%-41.2%)	21.0% (66)	22.4% (12.9%-36.0%)	30.2% (130)	31.0% (19.2%-45.9%)	
*Eastern Africa*		6.5% (48)	5.4% (3.3%-8.8%)	4.1% (13)	4.6% (2.4%-8.6%)	8.1% (35)	5.9% (3.1%-10.8%)	
*Southern Africa*		0.5% (4)	0.4% (0.1%-1.4%)	1.0% (3)	1.0% (0.3%-3.7%)	0.2% (1)	0.1% (0.0%-0.7%)	
**Migration duration***^a^*								.206
*< 2 years*		24.4% (170)	24.1% (20.1%-28.6%)	28.6% (83)	27.2% (20.1%-35.7%)	21.4% (87)	22.3% (17.9%-27.5%)	
*≥ 2 and < 10 years*		42.0% (293)	38.4% (34.4%-42.4%)	35.5%(103)	31.5% (23.0%-41.4%)	46.7% (190)	42.3% (36.9%-47.9%)	
*≥ 10 years or born in Belgium*		31.9% (234)	31.7% (26.2%-37.7%)	33.4% (104)	34.9% (26.2%-44.7%)	30.7% (130)	29.3% (22.9%-36.7%)	
**No health insurance**^b^		18.1% (135)	21.1% (16.6%-26.5%)	19.1% (60)	20.2% (14.8%-26.9%)	17.5% (75)	21.7% (15.4%-29.6%)	.756
**MIGRATION HISTORY**								
	**Prior migration in Africa***^a^* ^c^	23.2% (169)	24.7% (20.4%-29.6%)	17.2% (53)	16.1% (12.3%-20.9%)	27.5% (116)	29.8% (22.5%-38.2%)	.006*
	**Prior migration in Europe***^a^^d^*	30.4% (213)	31.7% (27.0%-37.0%)	26.7% (78)	28.6% (22.0%-36.2%)	33.1% (135)	33.6% (26.4%-41.6%)	.380
**MOBILITY**								
	**Travelled to Africa after migration**	44.6% (332)	47.1% (39.6%-54.7%)	43.9% (138)	49.3% (40.6%-58.0%)	45.1% (194)	45.8% (37.7%-54.2%)	.397
	**Travelled in Europe after migration**	56.3% (419)	56.9% (51.2%-62.4%)	54.8% (172)	54.3% (44.2%-64.1%)	57.4% (247)	58.4% (52.2%-64.3%)	.461

^a^ Missing variable (because ‘preferred not to answer) were excluded from analysis: migration duration (N = 697), prior migration in Africa (N = 730), prior migration in Europe (N = 700).

^b^ No health insurance of any kind: Belgian welfare system, health coverage via asylum center, health insurance in another European country or in an African country. This is a proxy for being undocumented (N = 743).

^c^ Participant lived longer than 6 months in another African country than his/her country of birth.

^d^ Participant lived longer than 6 months in other European country apart from Belgium. Those living abroad did not receive this question (N = 700).

* Significant difference between men and women at 5% level.

Sub-Saharan African migrants socializing in Antwerp city had diverse ethnic backgrounds, and reported different migration status and living situations. They originated from 35 different sub-Saharan African countries, with the largest groups being from Nigeria (24.9%), Democratic Republic of the Congo (16.9%), Cameroon (14.9%) and Ghana (12.0%). About one forth (24.1%) were recent migrants, and 37.5% were established migrants (living more than 10 years in Belgium or born there). One fifth (21.1%) did not have health insurance, which served as a proxy for an undocumented status. Many sub-Saharan African migrants had already migrated within Africa (24.7%) or Europe (31.7%) before arriving in Belgium and continued to be mobile after settling in Belgium.

About half (47.6%) of the sub-Saharan African migrants, significantly more men than women, were working or self-employed at the time of the study. Financial problems were prevalent, with almost two third (63.4%) reporting financial problems in the last year. Likewise, 8.8% did not have stable housing (i.e. homeless or living with friends).

### Sexual behavior and sexual health

Eighty-seven percent were sexually active in the last year. The majority preferred sexual partners of the opposite sex (92.9%) and of sub-Saharan African origin (73.0%). Sixty-two percent were currently married or in a relationship, and 69.4% of them were co-habiting. The median number of sexual partners in the last year was one, but significantly more men than women reported multiple partners and concurrency during the last year. Nearly forty percent (39.8%) of men in a relationship had other sexual partner(s) besides their stable partner in this time period. In addition, about one third (33.8%) assumed that their partner also had other sex partners in the last year.

Reduced sexual agency was common among both male and female sub-Saharan African migrants in Antwerp: 7.2% reported having sex with someone in exchange for gifts, food, money, papers or housing and emotional, 7.4% encountered physical violence by a partner and 1.8% were forced to have sex in the last year. One in ten (10,5%) women had been forced to have sex in their lifetime.

Thirty percent of sub-Saharan African migrants reported sexual encounters when travelling to Africa and within Europe, mostly with local sexual partners. Of them, two third (65.5%) reported using a condom at their last sexual encounter abroad. In general, 32.3% reported using a condom at last sexual contact. Four out of five (79.1%) reported that they are likely to use a condom with a future new partner, especially men have high condom intentions.

The reported degree of lifetime HIV testing was high (73.0%), and 40.4% of the total population were tested in the last year. Sixty percent reported to be aware of their last sexual partners’ HIV status. In the last year, 3.5% were diagnosis with a sexually transmitted infection (STI) other than HIV. Fourteen percent ever had an STI, significantly more men than women. More details can be found in Table 3 and Table 4.

**Table 3:** Sexual behavior of the sample (unweighted) and population estimates of sub-Saharan African migrants socializing in Antwerp city (weighted), stratified by gender.

	TOTAL		FEMALE		MALE		P-value	
	Sample	Population	Sample	Population	Sample	Population	Population	
	% (N = 744)	% (CI 95%)	% (N = 314)	% (CI 95%)	% (N = 430)	% (CI 95%)	
**In a relationship or married**	58.6% (419)	62.3% (56.5%-67.8%)	58.0% (174)	60.9% (52.7%-68.6%)	59.0% (245)	63.1% (55.6%-70.1%)	.664	
**Preferred sexual partner**							.939	
*Other sex*	93.3% (670)	92.9% (90.6%-94.8%)	91.7% (276)	92.8% (87.6%-96.0%)	94.5% (394)	93.0% (89.7%-95.3%)		
*Same sex or both*	2.9% (21)	3.7% (2.2%-6.3%)	3.7% (11)	3.5% (1.5%-8.1%)	2.4% (10)	3.9% (2.0%-7.3%)		
*Prefer not to answer*	3.8% (27)	3.3% (2.0%-5.4%)	4.7% (14)	3.6% (1.5%-8.7%)	3.1% (13)	3.1% (1.7%-5.5%)		
**Total number of sexual partners in the last year***^a^*							.009*	
*None*	12.4% (89)	10.8% (7.2%-15.8%)	14.0% (42)	12.3% (8.1%-18.4%)	11.3% (47)	9.9% (5.8%-16.3%)		
*1*	50.4% (362)	50.6% (45.8%-55.4%)	58.1% (175)	60.9% (53.6%-67.7%)	44.8% (187)	44.7% (38.5%-51.0%)		
*2*	17.7% (127)	19.7% (15.0%-25.4%)	15.6% (47)	17.1% (11.8%-24.1%)	19.2% (80)	21.2% (15.8%-27.8%)		
*3 to 6*	14.5% (104)	14.4% (11.4%-18.0%)	8.0% (24)	6.9% (3.8%-12.1%)	19.2% (80)	18.6% (14.3%-23.9%)		
*7 or more*	5.0% (36)	4.6% (2.7%-7.8%)	4.3% (13)	2.8% (0.7%-10.6%)	5.5% (23)	5.7% (3.2%-9.7%)		
**Origin last sexual partner***^a^*							.731	
*Sub-Saharan African*	73.3% (504)	73.0% (67.7%-77.7%)	75.4% (215)	74.3% (66.0%-81.2%)	72.4% (289)	72.2% (67.0%-76.8%)		
*Belgian*	21.9% (150)	21.9% (16.8%-28.0%)	19.6% (56)	21.3% (15.0%-29.4%)	23.6% (94)	22.2% (16.8%-28.7%)		
*Other*	4.4% (30)	5.1% (3.3%-7.9%)	4.9% (14)	4.3% (2.2%-8.4%)	4.0% (16)	5.6% (3.4%-9.2%)		
**Last sexual partner, casual***^a^*	21.7% (140)	19.9% (15.1%-25.7%)	12.3% (33)	10.5% (6.6%-16.3%)	28.5% (107)	25.2% (18.7%-33.0%)	.000*	
**Place last sex***^a^*							.527	
*Belgium*	83.6% (597)	81.9% (78.0%-85.2%)	85.3% (256)	84.4% (78.4%-89.0%)	82.4% (341)	80.5% (76.1%-84.2%)		
*European country*	6.4% (46)	7.9% (5.4%-11.3%)	6.3% (19)	7.1% (4.1%-11.9%)	6.5% (27)	8.3% (5.2%-13.0%)		
*Africa*	9.9% (71)	10.2% (8.2%-12.7%)	8.3% (25)	8.6% (5.2%-13.7%)	11.1% (46)	11.2% (8.3%-15.0%)		
**SEX AND MOBILITY**								
**Sexually active on African travels, lifetime***^b^*	20.0% (142)	21.8% (15.9%-29.1%)	10.7% (32)	14.6% (9.0%-22.8%)	26.8% (110)	26.0% (19.1%-34.1%)	.002*	
**Sexually active on African travels, in the last year***^b^*	9.2% (65)	11.4% (6.8%-18.5%)	3.4% (10)	6.0% (2.3%-15.1%)	13.4% (55)	14.5% (9.3%-22.1%)	.013*	
**Sexually active on European travels, lifetime**^c^	13.0% (92)	13.5% (10.7%-17.0%)	10.8% (32)	11.2% (7.4%-16.4%)	14.6% (60)	14.9% (11.3%-19.3%)	.201	
**Sexually active on European travels, in the last year**^c^	6.2% (44)	6.4% (4.2%-9.5%)	5.4% (16)	5.2% (2.3%-11.1%)	6.8% (28)	7.0% (4.1%-11.7%)	.524	
**Concurrent in the last year**^d^	33.0% (129)	33.7% (25.0%-43.7%)	23.9% (39)	23.2% (16.6%-31.4%)	39.5% (90)	39.8% (29.1%-51.7%)	.001*	
**TRANSACTIONAL SEX**								
**Transactional sex, lifetime***^a^* ^e^	11.9% (82)	10.8% (8.1%-14.1%)	14.0% (41)	12.3% (7.4%-19.7%)	10.3% (41)	9.9%(7.0%-13.7%)	.476	
**Transactional sex, in the last year***^a^* ^e^	7.1% (49)	7.2% (5.1%-10.0%)	8.2% (24)	7.4% (3.6%-14.4%)	6.3% (25)	7.1% (4.8%-10.4%)	.925	
**Paid for sex, lifetime***^a^*	18.5% (129)	19.9% (16.3%-24.1%)	2.7% (8)	4.3% (2.0%-9.0%)	29.9% (121)	28.8% (22.7%-35.8%)	.000*	
**Paid for sex, in the last year***^a^*	9.1% (63)	9.0% (6.2%-12.9%)	1.4% (4)	2.7% (0.9%-7.6%)	14.6% (59)	12.6% (7.9%-19.4%)	.010*	
**VIOLENCE**								
**Partner violence, lifetime***^a^* ^f^	15.4% (111)	14.8% (11.6%-18.7%)	17.3% (52)	16.4% (12.3%-21.4%)	14.0% (59)	13.9% (9.8%-19.3%)	.435	
**Partner violence, in the last year***^a^* ^f^	7.8% (56)	7.4% (5.0%-10.7%)	8.0% (24)	9.2% (5.6%-14.5%)	7.6% (32)	6.4% (3.8%-10.5%)	.270	
**Forced sex, lifetime***^a^* ^g^	8.0% (56)	6.6% (4.6%-9.3%)	13.1% (38)	10.5% (6.2%-17.2%)	4.4% (18)	4.4% (2.4%-7.8%)	.048*	
**Forced sex, in the last year***^a^* ^g^	2.1% (15)	1.8% (0.8%-3.8%)	3.1% (9)	1.8% (0.7%-4.6%)	1.5% (6)	1.8% (0.6%-5.6%)	.982	

^a^ Preferred not to answer and/or participant never had sex were excluded from analysis: total number of sexual partners in the last year (N = 718), origin last sexual partner (N = 684), type last sexual partner (N = 644), place last sex (N = 714), transactional sex (N = 691), paid for sex (N = 697), partner violence (N = 721), forced sex (N = 699).

^b^ Had sex with a stable or casual partner who live in Africa, when travelling to Africa after migration.

^c^ Had sex with a stable or casual partner who lives in another European country than their current country of residence, when travelling in Europe after migration.

^d^ Among participants in a relationship, had besides the stable partner, other sexual partner(s).

^e^ Had sex with someone in exchange for gifts, food, money, papers or housing.

^f^ Encountered emotional and/or physical violence by a partner.

^g^ Forced to have sex because somebody threatened them or used physical violence.

* significant difference between men and women at 5% level.

**Table 4:** Sexual health behavior of the sample (unweighted) and population estimates of sub-Saharan African migrants socializing in Antwerp city (weighted), stratified by gender.

	TOTAL		FEMALE		MALE		P-value
	Sample	Population	Sample	Population	Sample	Population	Population
	% (N = 744)	% (CI 95%)	% (N = 314)	% (CI 95%)	% (N = 430)	% (CI 95%)	
**CONDOM USE**							
**Low condom use intentions***^b^*	23.0% (171)	20.9% (16.3%-26.3%)	28.0% (88)	27.7% (20.8%-35.9%)	19.3% (83)	16.9%(12.1%-23.0%)	.011*
**No condom used at last sex**^a^	67.4% (458)	67.7% (61.0%-73.7%)	72.8% (206)	72.3% (62.6%-80.2%)	63.5% (252)	65.1% (58.6%-71.2%)	.108
**No condom used at last sex on African travel, lifetime**	7.3% (51)	8.3% (5.9%-11.4%)	4.1% (12)	5.4% (2.9%-9.9%)	9.7% (39)	8.3% (5.9%-11.4%)	.114
**No condom used at last sex on African travel, in the last year**	3.6% (25)	4.3% (2.8%-6.6%)	2.0% (6)	2.4% (0.9%-6.0%)	4.8% (19)	5.5% (3.3%-8.9%)	.125
**No condom used at last sex on European travel, lifetime**	4.1% (29)	4.6% (2.9%-7.0%)	3.7% (10)	3.9% (2.0%-7.4%)	4.4% (18)	4.9% (2.7%-8.9%)	.608
**No condom used at last sex on European travel, in the last year**	2.1% (15)	2.0% (1.0%-3.9%)	2.0% (6)	1.9% (0.7%-4.9%)	2.2% (9)	2.1% (0.8%-5.0%)	.928
**Alcohol used at last sex**	16.4% (118)	17.7% (13.4%-23.1%)	12.6% (38)	16.6% (10.4%-25.4%)	19.2% (80)	18.4% (12.9%-25.4%)	.722
**HIV TESTING**							
**HIV tested, lifetime**^a^	71.3% (513)	73.0% (68.3%-77.3%)	74.4% (224)	76.2% (69.3%-82.0%)	69.0% (289)	71.2% (64.3%-77.2%)	.272
**HIV tested, in the last year**^a^	41.3% (307)	40.4% (35.1%-45.9%)	44.3% (139)	44.0% (37.6%-50.6%)	39.1% (168)	38.4% (32.5%-44.5%)	.060
**Unaware of last partner’s HIV status**^a^	42.4% (302)	42.2% (37.4%-47.2%)	41.2% (121)	42.8% (35.3%-50.6%)	43.3% (181)	41.8% (35.9%-48.0%)	.544
**STI HISTORY**							
**STI diagnosis, lifetime**^a^ ^c^	12.9% (88)	14.4% (10.4%-19.6%)	9.6% (28)	9.9% (6.1%-15.7%)	15.3% (60)	17.1% (11.7%-24.3%)	.066
**STI diagnosis, in the last year**^a^ ^c^	4.1% (28)	3.5% (2.0%-5.9%)	4.1% (12)	3.4% (1.3%-8.3%)	4.1% (16)	3.5% (2.0%-6.3%)	.930
**HIV PREVALENCE**							
**HIV positive** ^d^	4.4% (32)	4.8% (2.7%-8.4%)	6.1% (19)	5.9% (3.4%-10.1%)	3.1% (13)	4.2% (1.6%-10.6%)	.533
**Undiagnosed HIV infection***^e^*	2.8% (20)	3.2% (1.4%-7.3%)	3.7% (11)	3.1% (1.5%-6.2%)	2.2% (9)	3.2% (0.9%-11.1%)	.964

^a^ Preferred not to answer and/or participant never had sex were excluded from analysis: Condom use at last sex (N = 680), HIV tested (N = 720), Aware of last partner’s HIV status (N = 712), STI diagnosis (N = 684).

^b^ Unlikely to use a condom with a future new sexual partner.

^c^ Self-reported diagnoses with an STI other than HIV.

^d^ Among participants with a valid oral fluid sample (N = 726).

^e^ Based on self-reported HIV-status.

* significant difference between men and women at 5% level.

### HIV prevalence

Thirty-two oral fluid samples were reactive to both tests resulting into an HIV prevalence estimation of 5.9% (95%CI:3.4%-10.1%) among women and 4.2% (95%CI:1.6%-10.6%) among men. Twenty among the 32 persons found HIV positive did not report their HIV status in the questionnaire, and therefore 65.2% (CI95%:32.4%-88.0%) were likely to be undiagnosed. Of the 12 persons, who reported their positive HIV status, ten were currently taking antiretroviral medication. Four HIV positive participants reported they were diagnosed with an STI in the last year, three of them were possibly undiagnosed. All HIV positive persons who reported unprotected sex when travelling in Europe did not report their HIV status. They had sex with a stable partner of sub-Saharan African origin who lives in another European country.

Factors associated with an HIV infection are presented in Table 5. In univariable analyses, HIV infection was associated with being unemployed (OR 2.59), no partner violence in the last year (OR 9.00), intention to use a condom with a future new partner (OR 3.85), unprotected sex when travelling within Europe in the last year (OR 6.53) and being unaware of their last sexual partners’ HIV status (OR3.70).

**Table 5:** Factors associated with HIV infection.

	HIV-prevalence	UNIVARIABLE		MULTIVARIABLE^*e*^
	% (95% CI)	OR (95% CI)	p-value	AOR (95% CI)
**Female** *(vs*. *male)*	5.9% (3.4%-10.1%)	1.44 (0.45–4.64)	.533	1.95 (0.62–6.15)
**Age** *(vs*. *18–24 years)*			.062	
*25–34 years*	3.3% (1.6%-6.8%)	5.28 (1.09–25.57)		3.40 (0.71–16.30)
*35–44 years*	6.4% (2.3%-16.8%)	10.45 (1.40–78.00)		4.80 (0.69–33.42)
*≥ 45 years*	9.1% (3.6%-21.0%)	15.23 (2.20–105.58)		14.34 (1.84–111.83)*
**Study setting** *(vs*. *public place)*			.105	
*Bar or parties of African organizations*	6.6% (3.4%-12.5%)	8.72 (2.00–37.97)		
*Church*	4.1% (1.3%-11.9%)	5.23 (0.92–29.59)		
*Meeting of African organization*	4.1% (1.9%-8.5%)	5.25 (1.16–23.74)		
*Other (shop*, *hair salon*, *library*, *asylum center)*	0.8% (0.1%-5.0%)	1.01 (0.11–9.67)		
**Living abroad** *(vs*. *residing in Belgium)*	9.5% (2.3%-31.5%)	2.22 (0.41–12.19)	.339	
**Educational level** *(vs*. *Continued education)*			.519	
*Secondary school*	3.9% (1.7%-8.8%)	1.03 (0.38–2.80)		
*Primary school or less*	6.7% (2.6%-16.2%)	1.83 (0.53–6.28)		
**Occupation** *(vs*. *employed or self-employed)*			.001*	
*Unemployed*	8.1% (4.3%-14.5%)	2.59 (1.28–5.27)*		2.46 (0.72–8.45)
*Full time student*	0.5% (0.1%-4.2%)	0.16 (0.02–1.50)		0.68 (0.05–8.80)
**Financial problems in the last year** *(vs*. *no)*			.904	
*Sometimes*	5.4% (3.0%-9.4%)	1.04 (0.41–2.64)		
*Most of the time*	4.5% (1.7%-11.2%)	0.87 (0.19–4.05)		
**Unstable housing** *(vs*. *stable housing)*	5.9% (2.3%-14.1%)	1.40 (0.42–4.72)	.574	
**Region of origin** *(vs*. *Western Africa)* ^a^			.589	
*Central Africa*	6.5% (2.4%-16.4%)	0.80 (0.22–2.88)		
*Eastern Africa*	5.1% (0.8%-25.5%)	2.33 (0.32–16.92)		
**Migration duration** *(vs*. *≥ 10 years or born in Belgium)*			.566	
*≥ 2 and < 10 years*	4.5% (2.3%-8.7%)	1.13 (0.57–2.21)		
*< 2 years*	5.7% (2.4%-13.0%)	1.44 (0.60–3.47)		
**No health insurance** *(vs*. *health insurance)*	2.4% (0.9%-6.4%)	0.42 (0.12–1.48)	.161	
**Prior migration in Africa** *(vs*. *no)*	4.4% (1.7%-11.1%)	0.91 (0.40–2.08)	.825	
**Prior migration in Europe** *(vs*. *no)*	4.8% (1.9%-11.8%)	1.18 (0.30–4.74)	.808	
**Travelled to Africa after migration***(vs*. *no)*	5.2% (2.9%-9.2%)	1.18 (0.64–2.17)	.587	
**Travelled within Europe after migration** *(vs*. *no)*	3.1% (1.7%-5.6%)	0.42 (0.12–1.51)	.170	
**Single** *(vs*. *in a relationship or married)*	4.1% (2.0%-8.3%)	0.77 (0.46–1.30)	.318	
**Preferred sexual partner** *(vs*. *other sex)* *^b^*			.678	
*Prefer not to answer*	6.7% (2.0%-20.1%)	1.35 (0.32–5.67)		
**Total number of sexual partners in the last year** *(vs*. *none)*			.141	
*1*	4.7% (2.7%-8.0%)	1.08 (0.27–4.33)		
*2*	6.2% (3.0%-12.2%)	1.44 (0.25–8.19)		
*3 to 6*	1.6% (0.3%-7.6%)	0.36 (0.04–3.56)		
*7 or more*	14.2% (3.6%-42.4%)	3.61 (0.42–31.25)		
**African partner at last sex** *(vs*. *Belgian partner)* **^c^**	5.8% (3.4%-9.9%)	1.69 (0.55–5.25)	.349	
**Last sexual partner, casual** *(vs*. *stable)*	4.5% (1.6%-12.5%)	0.97 (0.32–2.92)	.961	
**Place last sex** (*vs*. *Belgium)*			.096	
*Europe*	4.3% (1.2%-14.6%)	1.00 (0.23–4.35)		2.10 (0.40–11.09)
*Africa*	10.8% (3.3%-30.1%)	2.71 (1.03–7.10)		2.28 (0.68–7.67)
**Concurrent in the last year** *(vs*.*no)*	7.9% (2.7%-20.9%)	2.16 (0.50–9.42)	.288	
**Transactional sex, lifetime** *(vs*.*no)*	3.9% (1.7%-8.7%)	0.72 (0.23–2.31)	.572	
**Transactional sex, in the last year** *(vs*.*no)*	3.4% (1.1%-9.6%)	0.63 (0.17–2.33)	.477	
**Paid for sex, lifetime** *(vs*. *no)*	3.5% (1.1%-10.6%)	0.61 (0.17–2.25)	.449	
**Paid for sex, in the last year** *(vs*. *no)*	2.7% (0.5%-12.3%)	0.48 (0.09–2.70)	.387	
**Partner violence, lifetime** *(vs*. *no)*	1.6% (0.4%-5.5%)	0.29 (0.07–1.24)	.074*^d^*	
**Partner violence, in the last year** *(vs*. *no)*	0.6% (0.1%-4.6%)	0.11 (0.01–1.01)	.019*	0.08 (0.08–0.86)*
**Forced sex, lifetime** *(vs*. *no)*	1.9% (0.5%-7.5%)	0.29 (0.07–1.24)	.171	
**Forced sex, in the last year** *(vs*. *no)*	3.3% (0.4%-24.0%)	0.63 (0.06–6.09)	.678	
**Low condom use intentions** *(vs*. *high condom use intentions)*	1.5% (0.4%-5.2%)	0.26 (0.07–0.99)	.035*	0.22 (0.05–0.92)*
**No condom used at last sex** *(vs*. *condom used)*	4.0% (2.2%-7.1%)	0.62 (0.31–1.25)	.172	
**No condom used at last sex on African travel, lifetime** *(vs*.*no)*	6.0% (1.4%-21.5%)	1.21 (0.24–5.97)	.813	
**No condom used at last sex on African travel, in the last year** *(vs*. *no)*	9.9% (2.0%-37.2%)	2.14 (0.34–13.35)	.395	
**No condom used at last sex on European travel, lifetime** *(vs*.*no)*	13.1% (3.8%-36.7%)	3.11 (0.71–13.66)	.112	
**No condom used at last sex on European travel, in the last year** *(vs*. *no)*	24.1% (5.9%-61.6%)	6.53 (1.07–39.81)	.021*	5.47 (1.21–24.72)*
**Alcohol used at last sex** *(vs*. *no)*	8.4% (4.0%-16.8%)	2.10 (0.94–4.69)	.063	1.24 (0.43–3.58)
**Never tested for HIV** *(vs*. *ever)*	4.0% (1.2%-12.5%)	0.74 (0.25–2.22)	.588	
**HIV-test, in the last year** *(vs*. *≥ 1 year ago or never)*	7.9% (4.5%-13.6%)	2.86 (0.89–9.17)	.066	5.28 (1.83–15.29)*
**Unaware of last sexual partner’s HIV status** *(vs*. *aware)* **^a^**	7.8% (4.3%-13.7%)	3.70 (1.49–9.21)	.004*	5.16 (1.59–16.73)*
**Ever diagnosed with STI** *(vs*. *never)*	7.4% (2.6%-18.9%)	1.55 (0.64–3.75)	.325	
**STI diagnosis, in the last year** *(vs*. *≥ 1 year ago or never)*	12.4% (3.7%-34.0%)	2.69 (0.61–11.95)	.172	

^a^ Category ‘southern Africa’ has been excluded because there are no HIV positive cases who originate from that region.

^b^ The category ‘other sex or both sexes’ has been excluded because it was nonexistent among HIV positive migrants.

**^c^** The category ‘other’ has been dropped because none of the HIV positive cases answered this.

^d^ Since there is an overlap in cases with ‘partner violence, in the last year’, ‘partner violence, lifetime’ was excluded from multivariable analysis.

^e^ n = 593.

* significant difference between HIV-positive and HIV negative at 5% level.

In multivariable analyses, older age (45 years and older vs. 18 to 25 years), no partner violence in the last year, high intentions to use condoms, unprotected sex when travelling within Europe in the last year, being unaware of their last sexual partners’ HIV status and having had a recent HIV test (less than one year ago) remained independently and significantly associated with HIV-infection.

## Discussion

To the best of our knowledge this the first study assessing HIV prevalence in a representative sample of sub-Saharan African migrants living in a European city. We found an HIV prevalence of 5.9% among women and 4.2% among men of sub-Saharan African origin socializing in Antwerp city. The HIV epidemic among the communities of sub-Saharan African migrants in Europe therefore resembles the magnitude of the HIV epidemics in their home countries. Regardless of the recently reported decreasing numbers of new HIV diagnoses in surveillance [1,14], our findings indicate that the HIV burden is high in this population and may even increase further. Sixty-five percent of sub-Saharan African migrants living with HIV were likely undiagnosed. This is higher than the general European estimation of one in three [26], and elevates the risk of onward HIV transmission [27]. HIV infection was independently associated with being 45 years or older (compared to 18 to 25 years), high condom use intentions, recent HIV testing, unawareness of last sexual partners’ HIV status, no partner violence in the last year and sexual risk behavior when travelling within Europe in multivariable analysis. HIV positive sub-Saharan African migrants were 5.47 more likely to have had unprotected sex with a local partner when travelling in Europe compared to uninfected individuals. This demonstrates that African sexual networks are not limited to national borders and that HIV epidemics among sub-Saharan African migrant in different European countries are interlinked. Currently, HIV prevention strategies and policies differ between European countries [12,28], and for Belgium, even between regions. This local approach to HIV prevention does not match the needs of this mobile population, e.g. 56.9% of sub-Saharan African migrants in Antwerp travelled to another European country after settling in Belgium and 31.7% migrated within Europe before coming to Belgium. The lack of knowledge of local health services and rights constitutes a well-documented barrier for HIV prevention and testing uptake among sub-Saharan African migrants [12]. Consequently, an aligned European prevention approach is needed to effectively prevent HIV transmission among sub-Saharan African migrants across Europe.

At community level, our data show that sub-Saharan African migrants socializing in Antwerp are at increased risk of acquiring HIV due their sexual culture and hardship. The preference for African sexual partners, the existence of African sexual networks [16], and a culture of concurrency elevate the risk of HIV-transmission [3, 10]. Among those in a stable relationship, 33.7% had a sexual partner ‘on the side’ in the last year and 73.0% said their last sexual partner was of sub-Saharan African origin. In addition, many sub-Saharan African migrants in Antwerp are facing legal and financial problems: 21.1% were likely to be undocumented, 63.4% had financial problems in the last year and 8.8% lacked stable housing. The French ANRS PARCOURS study showed that this hardship increases the risk for HIV acquisition in France because it facilitates risky sexual relationships [29]. Our study partially corroborates this finding: we also found an association between being HIV-positive and unemployment (OR 2.59), but none between risky sexual behavior and HIV-status. The absence of an association between HIV-infection and risky sexual behavior is possibly due to the scale of our sample (i.e. 32 people living with HIV) and is therefore not generalizable. HIV-infection was independently associated to the absence of partner violence in the last year (AOR 0.09). This may be explained by relationship characteristics. A qualitative study among sub-Saharan African women living with HIV in Belgium showed that most women who disclosed their HIV status to their intimate partners received support and empathy from them [30]. Future research should focus on further identifying and describing the sub-groups within the sub-Saharan African migrant communities at increased risk of HIV acquisition in Europe in order to better tailor and target prevention interventions.

Efforts should also be made to better tailor existing HIV testing and prevention efforts. Our findings show that currently diagnostic and preventive opportunities are missed. While reported HIV testing uptake was high among sub-Saharan African migrants in Antwerp city (i.e. 73.0% reported lifetime testing, 40.4% in the last year), potentially 65.2% of those found positive were undiagnosed. This indicates that current HIV testing efforts might not reach the sub-groups at highest risk. 3.5% (N = 28) of sub-Saharan African migrants reported a STI diagnosis in the last year and 3 of them among people with an undiagnosed HIV-infection. This means that often an STI diagnosis did not automatically lead to detecting HIV regardless of STI-testing guidelines which recommend combined HIV/STI-testing [31].

Extensive efforts were made to ensure the study’s acceptance in the sub-Saharan African communities, participation and representativeness. We reached an acceptance rate of 60.2%, which is satisfactory considering the sensitivity of the research topic, the recruitment and data collection methods (eg. collection of oral fluid samples) and the general societal climate of pressuring migration laws. However, a selection bias cannot be excluded with regard to the study participants’ age and study setting. To include the diverse sub-groups of sub-Saharan African migrants, the sampling frame was developed in a systematic manner and included settings that covered most aspects of sub-Saharan African migrants’ social life in Antwerp. Yet, people who stay long term in facilities such as closed asylum centers, detention centers or hospitals were likely not reached. By including churches, shops and public settings likes streets, squares and parks we aimed at including people who are less often attending social venues. However, we cannot exclude underrepresentation of this sub-group and an effect on our HIV-prevalence estimates. Sub-Saharan African migrants living with HIV may isolate themselves due to fear for stigma, but do attend churches [30]. On the other hand, homebodies are less likely to meet new sexual partners and therefore may also be at reduced risk of acquiring HIV. This could have affected our results either way.

Interpreting the results in terms of numbers of sub-Saharan African migrants who are unaware of their HIV status requires caution. Underreporting, both intentionally and unintentionally, cannot be excluded. Regardless of the GIPA approach and extensive anonymity measures taken in our study, some respondents might not have been willing to disclose their HIV status in the questionnaire. Avoidant HIV coping strategies might have resulted in denial or ignorance of their HIV status [32] among some HIV positive study participants. In spite of this potential bias, we still conclude that the proportion of sub-Saharan African migrants being unaware of their positive HIV status found in this study is unacceptably high.

In conclusion, HIV prevalence among sub-Saharan African migrants in Antwerp city is high and the potential risk for future onwards HIV transmissions is elevated. An aligned European combination prevention approach [33] tailored to the needs of the sub-groups at highest risk (e.g. mobile and socially disadvantaged communities) is needed to prevent future HIV transmissions. Translating guidelines on integration of STI and HIV testing and counseling [31] into clinical practice and strengthened efforts to ensure HIV testing uptake among groups with a high risk for HIV acquisition should be prioritized. To be effective, they should be complemented with structural interventions at the policy level aiming at mitigating health related effects of economic hardship and ensuring access to health care and social rights [29].

## Supporting information

S1 File TOGETHER Project- database-PLOS One 2.sav. (SAV)
